# Placental adhesion disorder: magnetic resonance imaging features and a proposal for a structured report

**DOI:** 10.1590/0100-3984.2019.0037

**Published:** 2020

**Authors:** Thais Coura Figueiredo Agostini, Reginaldo Figueiredo, Gisele Warmbrand, Ulysses Santos Torres, Hanna Rafaela Ferreira Dalla Pria, Giuseppe D’Ippolito

**Affiliations:** 1 Departamento de Diagnóstico por Imagem da Escola Paulista de Medicina da Universidade Federal de São Paulo (EPM-Unifesp), São Paulo, SP, Brazil.; 2 Departamento de Anatomia e Imagem da Faculdade de Medicina da Universidade Federal de Minas Gerais (UFMG), Belo Horizonte, MG, Brazil.; 3 Grupo Fleury, São Paulo, SP, Brazil.; 4 Hospital São Luiz, São Paulo, SP, Brazil.

**Keywords:** Placenta accreta, Placenta previa, Magnetic resonance imaging, Placenta acreta, Placenta prévia, Ressonância magnética

## Abstract

Placental adhesion disorder encompasses the various types of abnormal placentation that occur when the chorionic villi penetrate the uterine wall. Placenta accreta has become more common, mainly because of the increasing rates of cesarean section. Although ultrasound is the first-line imaging modality for evaluation of the placenta, it plays a limited role in cases of posterior placenta accreta and inconclusive findings. In such cases, magnetic resonance imaging (MRI) is indicated, mainly because it is a more accurate means of identifying placental invasion of extrauterine structures in high-risk pregnant women. In this review article, we present the ten major and minor MRI features of placental adhesion disorder, as described in the international literature. In addition, we propose a template for structured reports of MRI examinations of the placenta. We have also devised a guided questionnaire in order to identify risk factors in patients scheduled to undergo such examinations, with the objective of facilitating the multidisciplinary treatment planning needed in order to minimize maternal morbidity and mortality.

## Abstract (pt)

A desordem de adesão placentária é um tipo de placentação anormal que ocorre quando há penetração das vilosidades coriônicas na parede uterina. O acretismo placentário tornou-se mais frequente, principalmente devido às taxas crescentes de cesarianas. A ultrassonografia é a modalidade de imagem de primeira linha para avaliação placentária, apresentando papel limitado nos casos de placenta posterior e achados duvidosos. Nesses casos, a ressonância magnética (RM) está indicada e é bastante eficaz, principalmente para identificar a disseminação extrauterina da placenta em gestantes de alto risco. Neste artigo de revisão apresentamos os dez principais sinais de desordem de adesão placentária pela RM descritos na literatura. Junto a isso, propomos um modelo de relatório estruturado e um questionário direcionado com o intuito de identificar os possíveis fatores de risco da paciente a ser submetida ao exame de RM da placenta, atendendo às expectativas dos especialistas envolvidos no planejamento do tratamento multidisciplinar necessário para minimizar a morbimortalidade materna.

## INTRODUCTION

Placental adhesion disorder (PAD), commonly known as placenta accreta, is a group of disorders resulting from a deficiency in the decidua basalis that causes the chorionic villi to penetrate the myometrium^(1)^. These disorders are classified according to the depth of uterine invasion by the trophoblastic tissue^(2,3)^. Placenta accreta vera is the least invasive type of PAD, characterized by an abnormal fixation of the placenta directly in the myometrium. Placenta increta is characterized by partial invasion of the myometrium by the placenta, whereas placenta percreta is characterized by full invasion of the myometrium, the placenta extending to or beyond the uterine serosa, in some cases extending to adjacent organs^(3,4)^. The major risk factors for placenta accreta are a previous cesarean section and placenta previa, less common risk factors including a history of conservative myomectomy, uterine artery embolization, curettage, or uterine rupture^(1^, as well as advanced maternal age^(5)^.

Research shows that the incidence of placenta accreta in the general population is approximately 0.9%, although that rate increases to 9% when there is concomitant placenta previa and can be as high as 35% when there is a combined history of placenta previa and cesarean section^(6)^. In patients who have undergone more than three cesarean sections, the risk of invasive placenta has been reported to be 67%^(5)^. Diagnosing PAD during the prenatal period is extremely important because the condition is often associated with postpartum hemorrhage, emergency hysterectomy, maternal morbidity, and maternal mortality^(1,5)^. Ultrasound, combined with the color Doppler technique, is the main diagnostic tool for evaluating an abnormal placenta. However, when the ultrasound findings are suspicious or inconclusive, as well as in cases of posterior placenta, magnetic resonance imaging (MRI) is recommended as a supplementary imaging technique^(7)^.

For the diagnosis of PAD, MRI has an overall sensitivity of 75-100% and a specificity of 65-100%, with negative and positive predictive values of 79-92% and 67.0-84.4%, respectively^(1)^. The method is useful for defining lateral or posterior placental extension to the periuterine or parametrial fat pads and for detecting invasion of the bladder^(5)^.

The objective of this article is to present the ten main MRI features of PAD, in order to facilitate their diagnosis. In addition, we propose a template for a structured report encompassing all of those features, as well as a questionnaire about the possible risk factors in patients who will undergo an MRI examination of the placenta.

## MRI PROTOCOL

It is preferable that MRI examinations be performed 1.5-T scanners, accompanied by body array coils^(4)^. 3-T equipment has the advantage of providing greater signal intensity, however, has the disadvantage of susceptibility artifacts and dielectric effects^(8)^.

To optimize the evaluation of the vesicouterine space, as well as to prevent motion artifacts and the patient discomfort caused by bladder overdistension during the examinations, the bladder should be only partially full^(2)^. The delivery of oxygen through a nasal cannula has proven to decrease fetal movement and to allow pregnant women to perform breath holds that are more prolonged and comfortable^(9)^.

For the evaluation of patients with PAD, MRI sequences are acquired in the axial, sagittal, and coronal planes^(6)^. The sequences employed include the following (Table 1): T2-weighted single-shot fast spin echo (SSFSE)/half-Fourier acquisition single-shot turbo spin-echo (HASTE)/single-shot turbo spin-echo (SSHTSE), to evaluate the uterine layers and placental architecture; T2-weighted fast imaging employing steady-state acquisition sequence (FIESTA)/true fast imaging with steady-state precession (TrueFISP)/balanced fast field echo (bFFE), to reduce breathing artifacts and to differentiate bands with low signal intensity from venous lakes; and T1-weighted gradient echo (GRE) sequences, to identify retroplacental hematoma. Sequences with high temporal resolution and a good contrast-to-noise ratio, which eliminate the underlying fetal motion, have made it possible to acquire placental images of high diagnostic quality^(7)^. A radiologist should monitor the test as it is being performed to determine whether additional sequences, such as coronal or sagittal oblique sequences, are necessary.

**Table 1:** Technical parameters.

					Slice	
		TR	TE	Flip	thickness	
MRI sequence*	Plane	(ms)	(ms)	angle	(mm)	Matrix
T2 HASTE/SSFSE/SSHTSE	Axial	Min	90	180	5-9	256 × 192
	Coronal					
	Sagittal					
2DTrueFISP/FIESTA/bFFE	Axial	6.71	3.26	65	6	256 × 256
	Sagittal					
Fat sat T2 FSE	Axial	5000	117	160	4	256 × 192
T1 GRE in/out	Axial	225	2.2/4.4	80	6	256 × 128
Fat sat 3D GRE T1	Axial	Min	Min	10/15	4.6	224 × 160

* All sequences were acquired in 1.5-T scanners manufactured by Siemens AG (Berlin, Germany), GE Healthcare (Milwaukee, WI, USA), or Philips Medical Systems (Best, The Netherlands). TR, repetition time; TE, echo time; T2, T2-weighted; 2D, two-dimensional; T1, T1-weighted; GRE, gradient-recalled echo; 3D, three-dimensional.

The first studies on the use of MRI for diagnosing PAD recommended the use of intravenous contrast to enhance the efficacy of the method^(10,11)^, although they presented no robust evidence to support that recommendation. A recent, important study involving a large cohort of pregnant women concluded that the use of paramagnetic contrast at any stage of pregnancy increases the risk of rheumatological and inflammatory effects, as well as of fetal and neonatal death^(12)^. Therefore, MRI scans for the investigation of PAD are currently performed without the use of intravenous contrast.

There is no consensus regarding the ideal gestational age at which to perform MRI in patients with suspected PAD. There is evidence that MRI has very low accuracy (< 50%, which is considered unacceptable) when it is performed before the 24th of gestation^(13)^. However, the accuracy is also low when it is performed after the 35th of gestation because of the accentuated myometrial thinning and the natural placental heterogeneity, limiting the use of these two MRI features. Therefore, it seems sensible to perform MRI between weeks 30 and 35, if possible. However, that suggestion may be subject to criticism. There are authors who recommend that MRI be performed between the 24th and 30th week of gestation, claiming that the placenta is naturally more homogeneous and the myometrium is thicker at that stage^(7)^.

## TEN MRI FEATURES OF PLACENTA ACCRETA

Numerous MRI features of PAD have been described in the literature. These features can be divided into major and minor abnormalities (Table 2), depending on whether the findings have a specificity above or below 80%. Such features include the following: abnormal uterine bulge; placental bulge; placental heterogeneity; placental protrusion into the cervical os or other adjacent structures; bands with low signal intensity (dark intraplacental bands) on T2-weighted images, accompanied by placental recess; dark bands on T2-weighted images; irregular placental-myometrial interface; myometrial thinning; abnormal placental vascularity; and subserosal hypervascularity.

**Table 2:** Major and minor MRI features of PAD.

Feature	Specificity	Sensitivity
Major features		
Abnormal uterine bulge	90.2%	79.1%
Placental bulge	90.3%	88.8%
Placental heterogeneity	87.7%	78.6%
Placental protrusion	82.4%	83.8%
Dark bands on T2-weighted images accompanied by placental recess	100.0%	56.0%
Minor features		
Dark placental bands on T2-weighted images	71.9%	87.9%
Irregular placental-myometrial interface	75.6%	92.0%
Myometrial thinning	6.5%	100.0%
Abnormal placental vascularity	37.6%	93.3%
Subserosal hypervascularity	73.1%	80.0%

### Major features Abnormal uterine bulge

**Figure 1:**
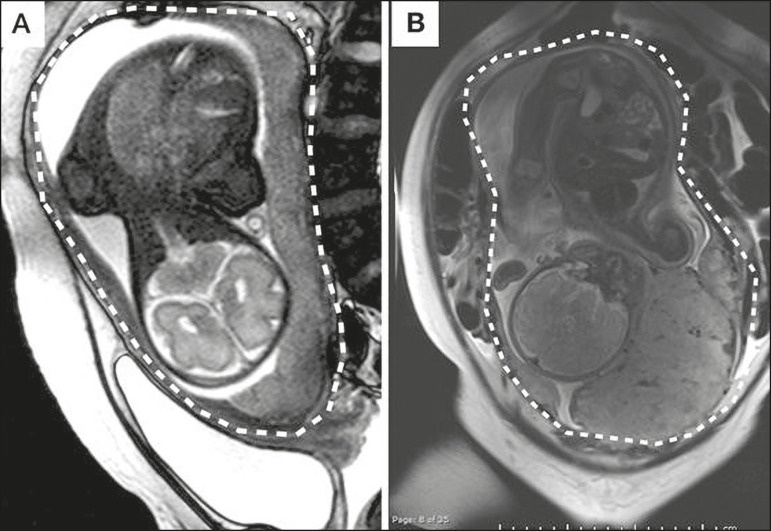
**A:** Sagittal T2-weighted MRI sequence showing a gravid uterus of normal appearance (inverted-pear shape) with no features of placenta accreta. **B:** Coronal T2-weighted MRI sequence showing a uterus with an hourglass shape, suggesting a diagnosis of placental adhesion disorder.

Abnormal uterine bulge can present as widening of the lower uterine segment. In coronal or sagittal MRI sequences, the uterus can have an hourglass-like appearance, rather than the usual inverted-pear shape^(6,14)^ (Figure 1).

### Placental bulge

**Figure 2:**
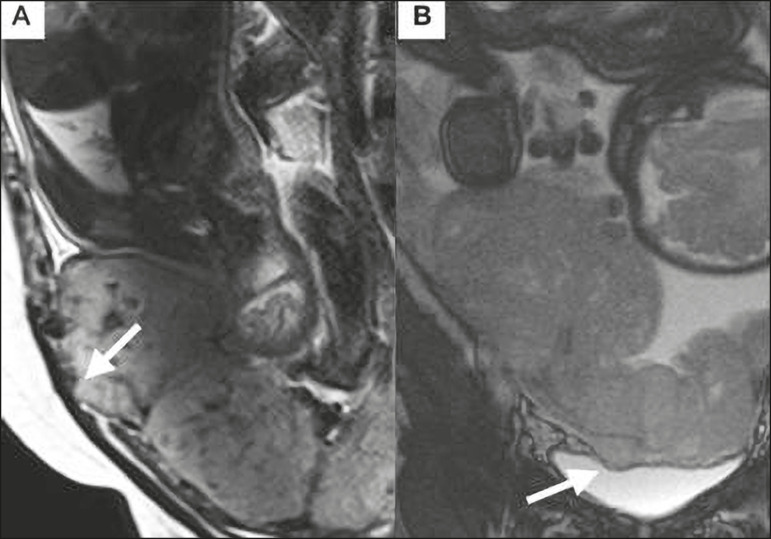
**A,B:** Sagittal T2-weighted MRI sequences showing a type II placental bulge (arrow in **A**) with a distorted uterine contour. There is also bulging of the posterior wall of the bladder caused by the placental bulge (arrow in **B**).

Placental bulge can be classified as one of two types, depending on the contour of the uterus. A type I placental bulge is defined as a slight bulge outward into the underlying myometrium when the uterine contour is intact and undistorted, whereas a type II placental bulge is defined as focal bulging with a distorted uterine contour and changes in the subjacent contour^(14)^, as shown in Figure 2.

### Placental heterogeneity

**Figure 3:**
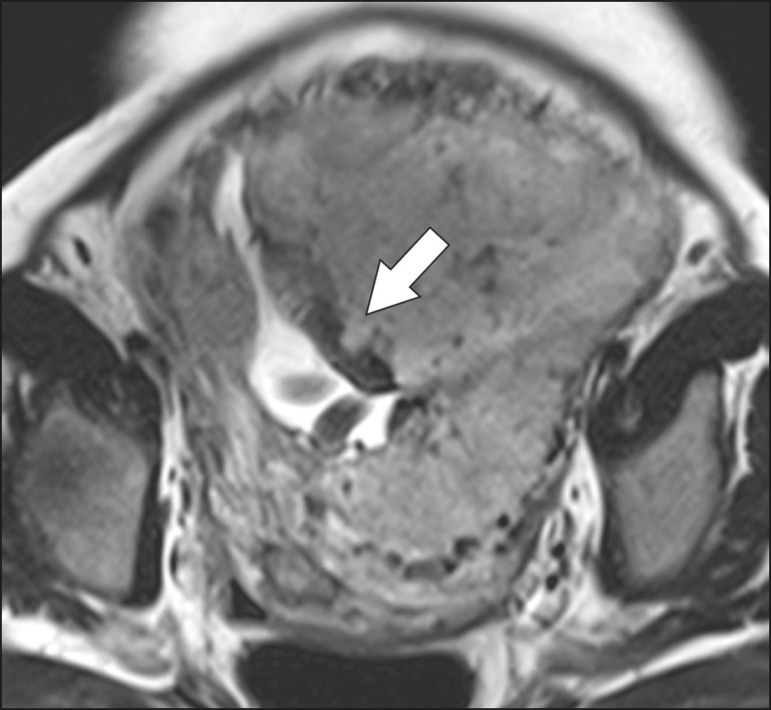
Sagittal T2-weighted MRI sequence showing placental heterogeneity, characterized by a diffuse signal and a dark intraplacental band (arrow).

A normal placenta is smooth and homogeneous, with intermediate signal intensity in T2-weighted MRI sequences^(1)^. As the pregnancy advances beyond the 32nd week, the placenta usually becomes heterogeneous, a phenomenon referred to as “heterogeneity of placental maturation”^(1)^. With regard to imaging parameters, the heterogeneity of the placenta remains subjective; the placenta can present characteristics such as intraplacental bands and flow voids, together with areas of placental infarction^(14)^, as shown in Figure 3. Up to 30% of patients without placenta accreta can have this feature. It is important to note that placental homogeneity should be evaluated in T2-weighted SSFSE/HASTE/SSHTSE sequences, given that there is a tendency to underestimate signal homogeneity when T2-weighted FIESTA/TrueFISP/bFFE sequences are employed^(11)^.

### Placental protrusion

**Figure 4:**
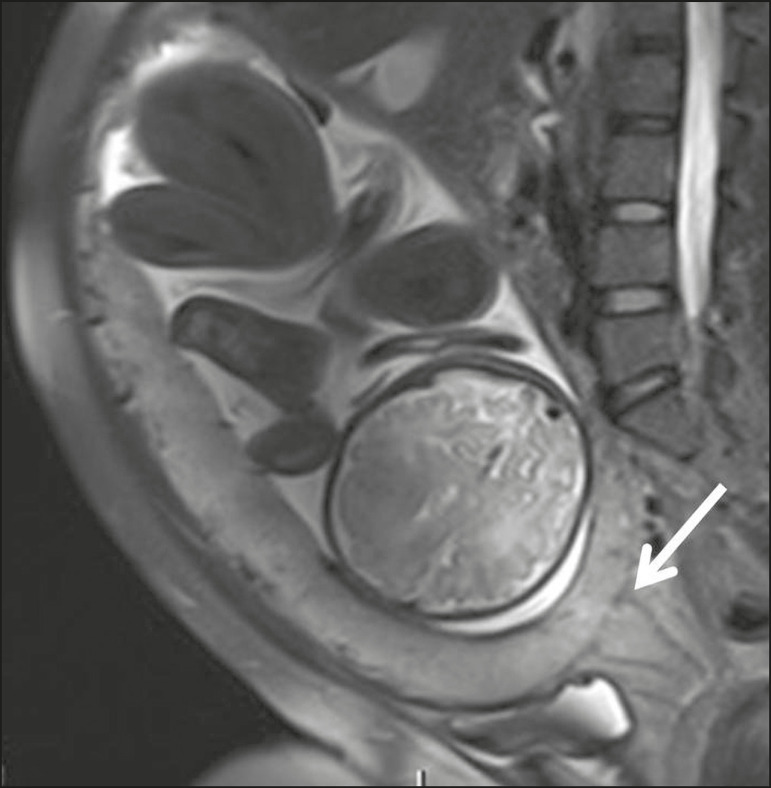
Sagittal T2-weighted MRI sequence showing placental protrusion into the internal cervical os.

Placental protrusion consists of myometrial invasion extending to the uterine serosa or crossing the internal cervical os and reaching adjacent structures^(6)^. In cases of placenta previa, placental protrusion through the internal cervical os is a reliable indication of placenta accreta (Figure 4). In cases of placenta percreta, MRI can show the placental tissue extending beyond the uterine serosa, all the way to the parametrium, bladder, or intestine^(14)^. To prevent false-positive results indicative of bladder invasion, the bladder should be partially full during image acquisition^(2)^.

### Dark bands on T2-weighted images accompanied by placental recess

**Figure 5:**
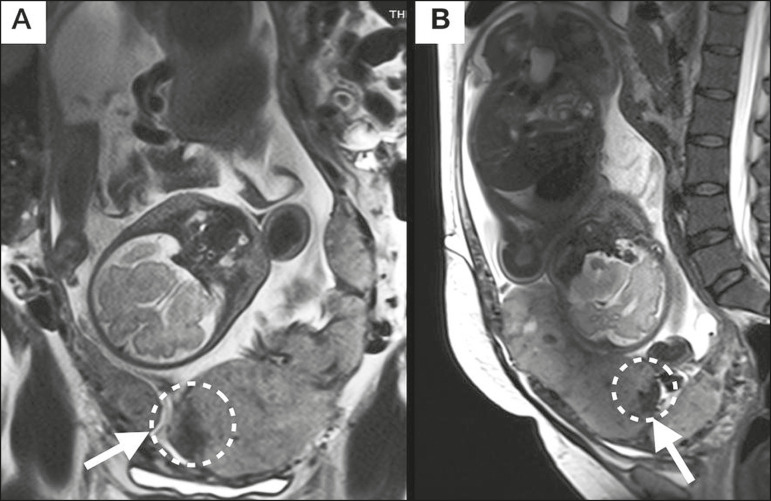
Coronal and sagittal T2- weighted MRI sequences (**A** and **B**, respectively) showing a placental recess (arrow) and dark bands (dotted circle).

**Figure 6:**
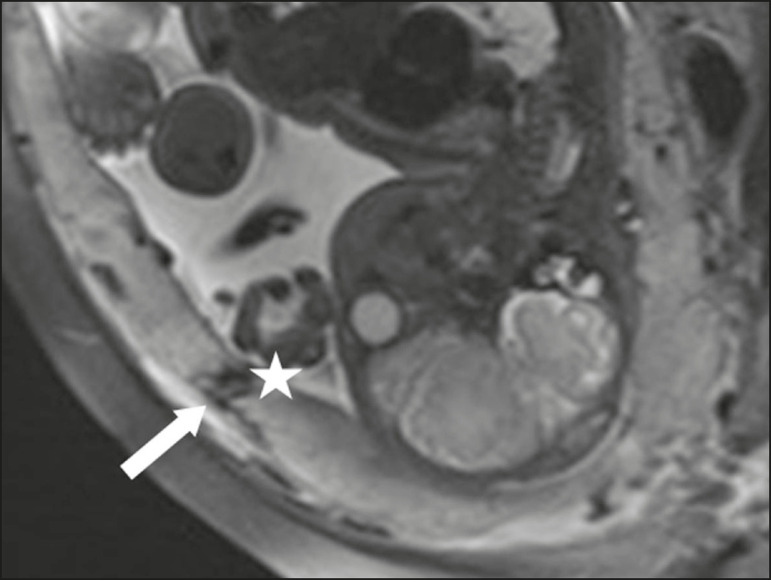
Sagittal T2-weighted MRI sequence showing placental recess and dark bands (star).

Placental recess is defined as placental deformity with contraction of the placental surface and of the outer rim of the uterus, which takes on a wedge-shaped appearance, with reduced thickness^(15)^. It is accompanied by a dark placental band on T2-weighted images (Figures 5 and 6), which is a minor feature.

### Minor features Dark placental bands on T2-weighted imaging

As previously mentioned, dark placental bands are nodular or linear areas of low signal intensity on T2-weighted images^(2)^. These bands often originate from the basilar plate on the maternal side of the placenta and have a longitudinal diameter > 2.0 cm, a thickness > 1.0 cm, and a random distribution^(6)^. These characteristics help differentiate the dark placental bands from the normal placental septa, which tend to be thin and smooth^(1)^. The bands are believed to represent areas of fibrin deposition in the placenta, possibly caused by frequent hemorrhage and infarction. There is a correlation between the increase in the volume of the band and the degree of placental invasion^(6)^. If the placenta is homogeneous and has no bands, it is unlikely that the placenta has become invasive (Figures 5 and 6).

### Irregular placental-myometrial interface

**Figure 7:**
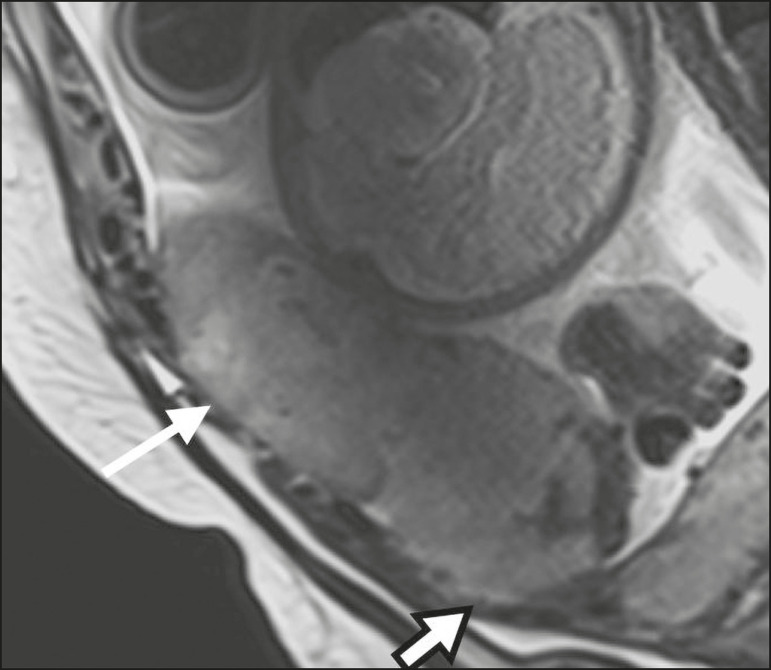
Sagittal T2-weighted MRI sequence showing a normal thin layer with low signal intensity between the myometrium and the placenta (white arrow) and an irregular placental-myometrial interface (outlined arrow).

The uterine-placental interface is the line of demarcation between the placenta and the uterus. The complex into which this interface is inserted consists of three parallel layers: the innermost layer (the decidua); the middle layer (the myometrium); and the outermost layer (the uterine serosa). On T2-weighted MRI scans, the signal intensity is low in the decidua, intermediate in the myometrium, and low in the uterine serosa^(6)^. Focal thinning or a defect in the uterine-placental interface, particularly in the decidua (Figure 7), is considered a significant predictor of an invasive placenta^(6)^. However, that feature can be nonspecific in cases of advanced gestational age and thinning at the site of a previous cesarean section.

### Myometrial thinning

**Figure 8:**
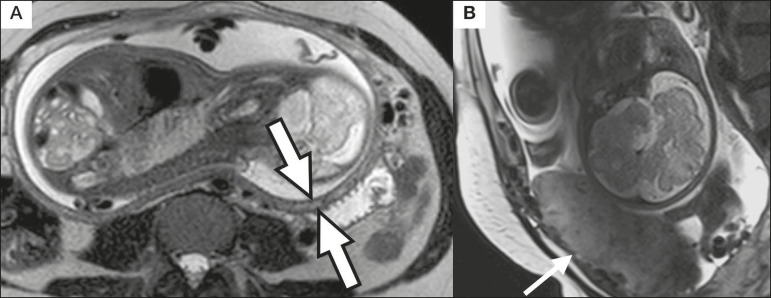
**A:** Axial T2-weighted MRI sequence showing a normal trilaminar appearance of the myometrium, represented by the middle layer with high signal intensity whereas the outer and inner layers have low signal intensity (arrows). **B:** Sagittal T2-weighted sequence showing myometrial thinning with indistinct layers (arrow).

Myometrial thinning is the loss of the normal trilaminar appearance of the myometrium, with preservation of its outer layer^(14)^, as depicted in Figure 8.

### Abnormal placental vascularity

**Figure 9:**
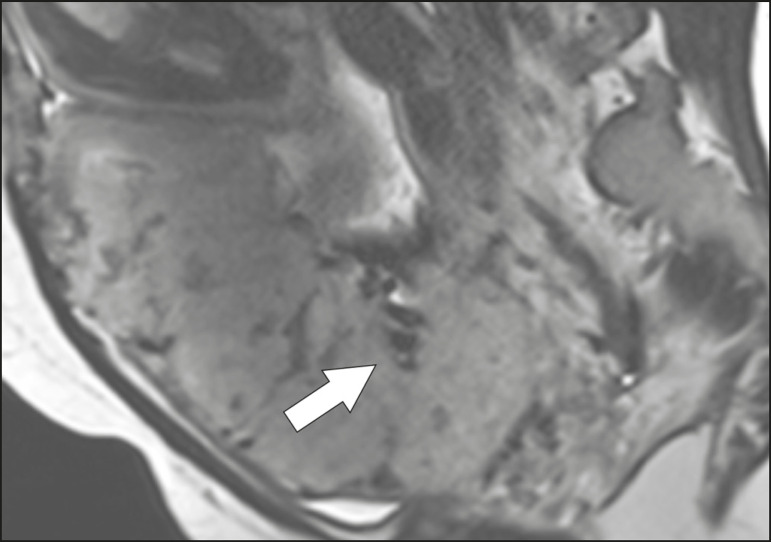
Sagittal T2-weighted MRI sequence showing complete placenta previa accompanied by disorganized placental vascularity and vessels with calibers going up to 9 mm (arrow).

Abnormal placental vascularity consists of disorganized, tortuous, dilated intraplacental vessels with calibers > 0.6 cm, usually located next to dark intraplacental bands on T2-weighted images. There is a probable correlation between the extent of abnormal placental vascularity and the degree of invasion, the most bizarre vasculature patterns existing in cases of placenta percreta^(1)^. The identification of abnormal vascularity relies on the comparison between T2-weighted images HASTE, SSFSE and SSHTSE sequences, in which the vessels show no flow or flow voids, and FIESTA/TrueFISP/bFFE sequences, in which the vessels return high signal intensity^(6,14)^, as illustrated in Figure 9.

### Subserosal hypervascularity

**Figure 10:**
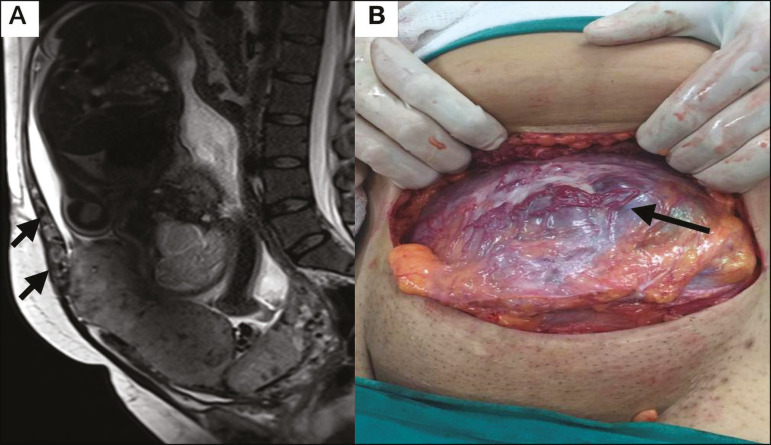
**A:** Sagittal T2-weighted image showing subserosal hypervascularity of the uterus with flow voids (arrows). **B:** Intraoperative appearance.

Subserosal hypervascularity consists of tortuous, compacted vessels throughout the uterine serosa, in the lower uterine segment. On axial images, subserosal hypervascularity appears as vessels with no flow or flow voids^(14)^, as shown in Figure 10.

## STRUCTURED REPORT

**Figure 11:**
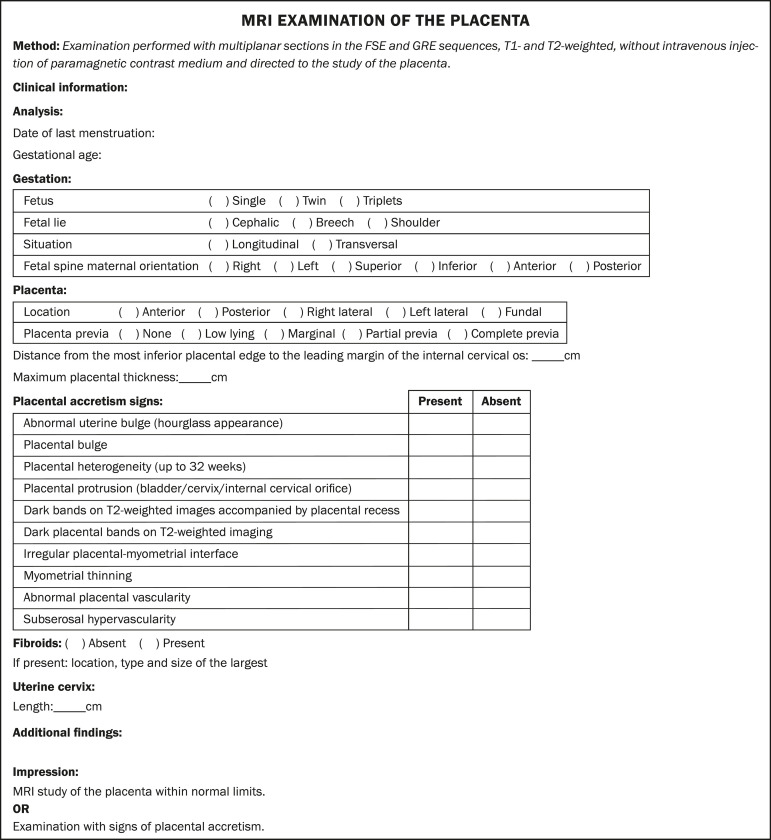
Template for a structured report of MRI examinations of the placenta with a diagnostic hypothesis of placenta accreta.

A structured report offers opportunities for improving the quality of imaging methods reporting. By using standardized terminology, imaging findings can be communicated with greater clarity and objectivity, which can provide significant benefits for the treatment and monitoring of a disease^(16)^. At our center, we devised a structured report encompassing the main features of PAD described in the literature, in addition to the basic imaging parameters (Figure 11). We also devised a questionnaire that addresses possible risk factors in patients scheduled to undergo MRI examination of the placenta (Table 3).

**Table 3:** Questionnaire for patients scheduled to undergo MRI examination of the placenta.

1) How many times have you been pregnant? 2) How many cesarean sections have you had? 3) Have you ever had a curettage, a myomectomy, or a fibroid embolization? 4) Do you have any urinary or intestinal issues?

## CONCLUSION

Placenta accreta has become more common, mainly because of increasing rates of cesarean section^(7)^. Ultrasound continues to be the first-line imaging modality for evaluating the placenta, however, in cases of posterior placenta and inconclusive findings^(6)^ , MRI has shown satisfactory accuracy,^(8,17)^ which justifies its use to identify extrauterine spread of the placenta, playing an important role in the diagnosis of abnormal placenta in high-risk pregnant women^(5)^. Abnormal uterine bulge, placental bulge, placental heterogeneity, placental protrusion into the cervical os or into other adjacent structures, and dark intraplacental bands of T2-weighted images accompanied by placental recess have high specificity and are classified as major features.

We propose strict adherence to the protocol, along with the use of a guided questionnaire and a structured report. The objectives should be to increase the efficacy of MRI and to facilitate the multidisciplinary treatment planning needed in order to improve the care provided to patients with an invasive placenta, as well as to minimize maternal morbidity and mortality.

## References

[1] Cuthbert F, Vinas MT, Whitby E (2016). The MRI features of placental adhesion disorder-a pictorial review. Br J Radiol.

[2] Baughman WC, Corteville JE, Shah RR (2008). Placenta accrete: spectrum of US and MR imaging findings. Radiographics.

[3] Bour L, Placé V, Bendavid S (2014). Suspected invasive placenta: evaluation with magnetic resonance imaging. Eur Radiol.

[4] Derman AY, Nikac V, Haberman S (2011). MRI of placenta accrete: a new imaging perspective. AJR Am J Roentgenol.

[5] Bourgioti C, Zafeiropoulou K, Fotopoulos S (2018). MRI features predictive of invasive placenta with extrauterine spread in high-risk gravid patients: a prospective evaluation. AJR Am J Roentgenol.

[6] Azour L, Besa C, Lewis S (2016). The gravid uterus: MR imaging and reporting of abnormal placentation. Abdom Radiol (NY).

[7] Kilcoyne A, Shenoy-Bhangle AS, Roberts DJ (2017). MRI of placenta accreta, placenta increta, and placenta percreta: pearls and pitfalls. AJR Am J Roentgenol.

[8] Rahaim NSA, Whitby EH (2015). The MRI features of placental adhesion disorder and their diagnostic significance: systematic review. Clin Radiol.

[9] Shetty MK, Dryden DK (2015). Morbidly adherent placenta: ultrasound assessment and supplemental role of magnetic resonance imaging. Semin Ultrasound CT MR.

[10] Levine D, Hulka CA, Ludmir J (1997). Placenta accrete: evaluation with color Doppler US, power Doppler US, and MR imaging. Radiology.

[11] Lax A, Prince MR, Mennitt KW (2007). The value of specific MRI features in the evaluation of suspected placental invasion. Magn Reson Imaging.

[12] Ray JG, Vermeulen MJ, Bharatha A (2016). Association between MRI exposure during pregnancy and fetal and childhood outcomes. JAMA.

[13] Horowitz JM, Berggruen S, McCarthy RJ (2015). When timing is everything: are placental MRI examinations performed before 24 weeks' gestational age reliable?. AJR Am J Roentgenol.

[14] Chen X, Shan R, Zhao L (2018). Invasive placenta previa: placental bulge with distorted uterine outline and uterine serosal hypervascularity at 1.5T MRI - useful features for differentiating placenta percreta from placenta accreta. Eur Radiol.

[15] Sato T, Mori N, Hasegawa O (2017). Placental recess accompanied by a T2 dark band: a new finding for diagnosing placental invasion. Abdom Radiol (NY).

[16] Schwartz LW, Panicek DM, Berk AR (2011). Improving communication of diagnostic radiology findings through structured reporting. Radiology.

[17] Familiari A, Liberati M, Lim P (2018). Diagnostic accuracy of magnetic resonance imaging in detecting the severity of abnormal invasive placenta: a systematic review and meta-analysis. Acta Obstet Gynecol Scand.

